# Pesticide exposure and the microbiota-gut-brain axis

**DOI:** 10.1038/s41396-023-01450-9

**Published:** 2023-06-16

**Authors:** Rie Matsuzaki, Eoin Gunnigle, Violette Geissen, Gerard Clarke, Jatin Nagpal, John F. Cryan

**Affiliations:** 1 APC Microbiome Ireland, University College Cork, T12 YT20 Cork, Ireland; 2 Department of Anatomy and Neuroscience, University College Cork, T12 YT20 Cork, Ireland; 3 Department of Environmental Sciences, Wageningen University & Research, 6700AA Wageningen, The Netherlands; 4 Department of Psychiatry & Neurobehavioural Sciences, University College Cork, T12 YT20 Cork, Ireland; 5 School of Pharmacy and Department of Pharmacology & Therapeutics, University College Cork, T12 YT20 Cork, Ireland

**Keywords:** Microbiology, Environmental sciences, Microbiome

## Abstract

The gut microbiota exist within a dynamic ecosystem shaped by various factors that includes exposure to xenobiotics such as pesticides. It is widely regarded that the gut microbiota plays an essential role in maintaining host health, including a major influence on the brain and behaviour. Given the widespread use of pesticides in modern agriculture practices, it is important to assess the long-term collateral effects these xenobiotic exposures have on gut microbiota composition and function. Indeed, exposure studies using animal models have shown that pesticides can induce negative impacts on the host gut microbiota, physiology and health. In tandem, there is a growing body of literature showing that the effects of pesticide exposure can be extended to the manifestation of behavioural impairments in the host. With the increasing appreciation of the microbiota-gut-brain axis, in this review we assess whether pesticide-induced changes in gut microbiota composition profiles and functions could be driving these behavioural alterations. Currently, the diversity of pesticide type, exposure dose and variation in experimental designs hinders direct comparisons of studies presented. Although many insights presented, the mechanistic connection between the gut microbiota and behavioural changes remains insufficiently explored. Future experiments should therefore focus on causal mechanisms to examine the gut microbiota as the mediator of the behavioural impairments observed in the host following pesticide exposure.

## Introduction

The diverse consortia of microorganisms that reside in the gut, known as the gut microbiota, is fundamental to host metabolism, intestinal homeostasis as well as brain health and behaviour [1–4]. The gut microbiome is a dynamic ecosystem in which complex relationships and sensitivities exist, which ultimately determine the overall health of the host [5, 6]. Like any ecosystem, the gut microbiota are shaped by numerous factors namely host genetics, diet, drug exposure, physiology and environment. In particular, environmental perturbations that the gut microbiota experience are important in shaping community structure and functional outcomes. Hence, it is essential to understand the effects of specific chemicals, such as pesticides and other environmental pollutants, which are considered xenobiotics to the human body (i.e., chemical substances that are foreign to animal life) [7, 8].

### Microbiota-gut-brain axis

The mutualistic relationship between the brain and the gut microbiota is essential for maintaining the healthy mental state of the host, including brain function [1, 4, 9]. Indeed, the gut microbiota and central nervous system (CNS) communicate bi-directionally via the microbiota-gut-brain axis [1]. Out of 11 classified phyla, the human gut microbiota are dominated by four phyla: *Actinomycetota* (formerly known as *Actinobacteria*), *Bacteroidota* (formerly known as *Bacteroidetes*), *Bacillota* (formerly known as *Firmicutes*) and *Pseudomonadota* (formerly known as *Proteobacteria*) [10, 11]. The composition and diversity of the gut microbiota influence its various functional outcomes such as metabolism, barrier integrity and trophic functions [12]. There are several routes proposed as the main active communication pathways between the gut and the brain, including the neuroendocrine system, the vagus nerve and hypothalamic–pituitary–adrenal (HPA) axis routes. While some xenobiotics may influence the host directly by de/activating the chemical(s), others may impact the host via indirect routes. For instance, microbially produced neuromodulators in the gut, such as short chain fatty acids (SCFA’s) and neurotransmitters (e.g., serotonin, gamma-aminobutyric acid (GABA)), can alter the signals to the brain by disrupting the communication via the enteric nervous system [7]. Another key contributor to this crosstalk is the immune system, which plays a key role in maintaining the integrity of the intestinal barrier [1].

Given that xenobiotics can influence the microbiota-gut-brain axis [7], it is timely to consider that the mode of action of some pesticides includes disrupting this communication by modifying microbial viability or function. In fact, exposure to pesticides have been shown to disrupt gut microbiota composition [13, 14] while also found to have negative impacts on cognitive processes in humans [15, 16]. It remains to be resolved however, if changes in gut microbiota composition and function from microbe-pesticide interactions are the driving factor for the negative behavioural changes. Therefore, in this review, we evaluate the emerging evidence that there is a causal relationship between pesticide exposure and behavioural alterations via the microbiota-gut-brain axis. We propose the gut-brain axis as a potential avenue for further understanding the overall effects of pesticides on gut and brain health.

### Pesticides

Herbicides, insecticides and fungicides are three main classes of pesticides most widely used in agricultural and domestic situations leading to greatest human and environmental exposures. Each group can be categorised in numerous ways, such as the target organism(s), toxicity, chemical composition, mode of entry and mode of action (Table 1) [17, 18]. Commercial formulations are made up of active compounds and inert ingredients, such as emulsifiers, solvents and fragrances. However, differences in these formulations as well as chemical structure complicates our ability to measure impacts accurately and consistently on animal physiology and gut microbial ecosystem. For instance, different effects on the gut microbiota in animals have been recorded with the herbicide glyphosate on its own, in the commercial product Roundup (Bayer) and its metabolite aminomethylphosphonic acid (AMPA) (Supplementary Table 1) [19–21]. Additional factors such as dosage, administration duration and host profile (e.g., age, sex and exposure history) can also explain the different impacts on the gut microbiota profile [22]. Nevertheless, there is a large body of evidence to suggest a role of pesticides present in food, water and environment (e.g., air) in shaping the gut microbiota with a potential link to behaviour.

**Table 1:** Overview of active ingredients used for pesticides with its characteristics.

Active ingredients	Category	Chemical classes	Chemical structure	Mode of action and potential consequences
Aldicarb	Insecticide	Carbamates		AChE inhibitors; neuromuscular interference
Atrazine	Herbicide	Triazines		Photosystem II Inhibitors; endocrine system disruption
Chlorpyrifos	Insecticide	Organophosphates		AChE inhibitors; neuromuscular interference
Cypermethrin	Insecticide	Pyrethroids/Pyrethrins		Sodium channel modulators; nervous system signalling interference
Glufosinate Ammonium	Herbicide	Phosphinic acids		Glutamine synthetase inhibitors; glutamate-glutamine homeostasis interference
Glyphosate	Herbicide	Glycine		Enolpyruvyl shikimate-3-Phosphate (EPSP) synthase inhibitors; aromatic amino acid nutrition disruption
Imazalil	Fungicide	Imidazoles		Ergo/sterol biosynthesis demethylation inhibitors; membrane integrity and fluidity interference
Imidacloprid	Insecticide	Neonicotinoids		nAChR competitive modulators; neuromuscular interference
Lindane	Insecticide	Organochlorines		GABA-gated chloride channel antagonists; nervous system signalling interference
Nitenpyram	Insecticide	Neonicotinoids		nAChR competitive modulators; neuromuscular interference
Penconazole	Fungicide	Triazoles		Ergo/sterol biosynthesis demethylation inhibitors; membrane integrity and fluidity interference
Permethrin	Insecticide	Pyrethroids/Pyrethrins		Sodium channel modulators; nervous system signalling interference
Propamocarb	Fungicide	Carbamates		Lipid synthesis inhibitor; cell membrane permeability and fatty acids disruption
Quinalphos	Insecticide	Organophosphates		AChE inhibitors; neuromuscular interference
Thymol	Fungicide	Phenols		Multiple modes of action proposed including cell membrane disruptors

*AChE* acetylcholinesterase, *nAChR* nicotinic acetylcholine receptor.

Data on mode of action derived from - Herbicide Resistance Action Committee (HRAC; https://www.hracglobal.com), Weed Science Society of America (WSSA; Weed Science Society of America (WSSA; https://wssa.net), Insecticide Resistance Action Committee (IRAC; https://irac-online.org), Fungicide Resistance Action Committee (FRAC; https://www.frac.info) Chemical structures were drawn using BIOVIA, Dassault Systèmes, BIOVIA Draw 2022, San Diego: Dassault Systèmes, 2023.

### Pesticide exposure on the host

**Fig. 1:**
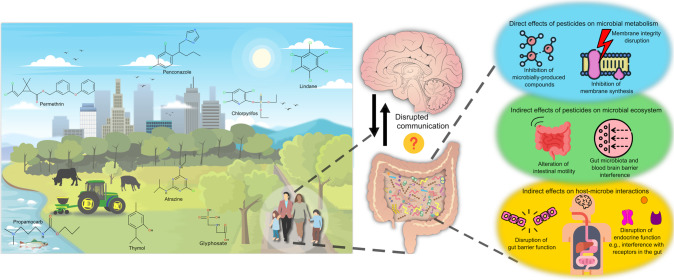
Proposed mechanisms of environmental pesticides impacting the microbiota-gut-brain axis. The residue of pesticides in the environment (air, soil, water and food) can enter the body of the host leading to disrupted communication between the gut and the brain. While the mechanisms are still being uncovered, some of the potential routes include, solely or in combination of, direct effects on microbial metabolism, indirect effects of pesticides on microbial communities in the gut and indirect effects on host-microbe interactions, which may explain the disrupted communication as seen from behavioural impairments. The icons used in this figure were designed by adriansyah, Flat icons, Freepik and Kalashnyk on https://www.flaticon.com/ and the chemical structure was drawn using BIOVIA, Dassault Systèmes, BIOVIA Draw 2022, San Diego: Dassault Systèmes, 2023.

The impact of pesticides on the gut microbiota are only now beginning to be resolved, considering that up until recently many widely used chemical pesticides were deemed to be safe for domestic and agricultural applications. The extensive, dependent usage of pesticides has resulted in very high concentrations reaching far beyond their intended target levels in our soil and water (Fig. 1). For instance, a large-scale study investigating >300 agricultural topsoil samples from across the European Union found that 80% of the tested soils contained pesticide residues [23]. Strikingly, residues of numerous pesticide mixtures that include glyphosate and its metabolites have also been detected in vegetables and fruits [24, 25]. These residual chemicals can enter the body via inhalation, ingestion or dermal absorption, which significantly increases the risk to the host [26].

Conveniently, the body is equipped with mechanisms to metabolise xenobiotics. For example, the biotransformation of pesticides by the microbiota has long been studied [27, 28]. The ability of microbiota to degrade and/or detoxify pesticides directly affects its bioavailability in the environment and within the exposed individual. However, when it comes to knowledge on the effects of the gut microbiota on the bioavailability of pesticides, while the topic is understudied, some findings do indicate a role for  gut microbiota in pesticide release and metabolism [29]. Additionally, the liver is the prime organ for xenobiotic metabolism in vertebrates, using enzymes such as cytochrome P_450_ [30]. However, the gut microbiota can still be directly exposed to many ingested xenobiotics before and after they reach the liver, causing unwanted health consequences depending on its status of biological availability [31]. These xenobiotics then could induce a wide variety of direct effects (e.g., shifts in microbial structure and function) and indirect effects (altered gut microbial metabolites shifting the expression and function of key gut and liver enzymes) [7, 32]. As such, gut-microbiota-mediated effects from exposure to pesticides may be an overlooked pathway impacting host health and behaviour [7, 14].

### Potential mechanisms for gut microbiota mediated effects on host upon pesticide exposure

Generally, the principles underpinning the postulated mechanisms can be divided into three broad categories (Fig. 1). The first being the direct effects of pesticides on microbial metabolism/physiology. Evidently, pesticides can cause direct toxic effects on microbial communities such as inhibition of microbially produced compounds (e.g., glyphosate mediated inhibition activity of EPSPS enzyme in shikimate pathway [33]) and inhibition of membrane synthesis (e.g., triazine mediated cell membrane disruption in photosynthetic microorganisms, conazoles mediated ergosterol synthesis disruption in fungal cell membrane [34]). This disruption in this microbe-host mutual relationship via the changes in the microbially produced compounds interferes with biosynthetic pathways for vitamins and aromatic amino acids in nutrition-based mutualism, which is crucial for host health [35]. Another important consideration is the antimicrobial resistance that could result from prolonged pesticide exposure. Indeed, the persistence of pesticides in the environment can promote pesticide-degrading gut microbiota to enhance their antimicrobial resistant characteristic to build a tolerance against it [36]. However, much of this understanding has been driven by studies in environmental systems like water and soil, with information on gut microbiota communities comparatively lacking.

The second avenue for the mechanistic explanation is indirect effects of pesticides on the gut microbial ecosystem. Indeed, pesticides can shift the physical and biochemical characteristics of the gut environment, which may suppress some microbial species while stimulating growth and survival of others, for example opportunistic pathogens in bees [37]. The imbalance of gut microbiota community structure due to individual or community-level changes can lead to irregular intestinal motility or gut/brain barrier perturbations, which are crucial factors for a healthy gut environment [38, 39].

The third category is indirect effects on outcomes of host-microbe interactions. An important facet of this is the implications of pesticides on host barrier function. Indeed, the gastrointestinal tract is a fundamental physical and biological barrier and is a primary site of exposure to toxic agents like pesticides, with the gut microbiota responsible for the development, maturation and regulation of the gastrointestinal tract [40]. The blood brain barrier, which protects and lines the inner surfaces of the blood vessels inside your brain, is also vulnerable to single or repeated exposure of certain pesticides [41]. Whether this is primarily mediated through alterations in gut microbiota structure and function is still yet to be understood. Other indirect effects on host-microbe interactions includes disrupted endocrine function. Indeed, organochlorine pesticides with endocrine disrupting capacity have also been related to alterations in gut microbiota [42].

The aim of this review is to first summarise currently known impacts of common pesticides on behaviour and gut microbiota in various animal models. Next, we will discuss the current research and future avenues for identifying causes and mechanisms underpinning negative implications of pesticide exposure from the microbiota-gut-brain axis perspective.

## Pesticide exposure and the gut-brain axis: the current paradigm

**Fig. 2:**
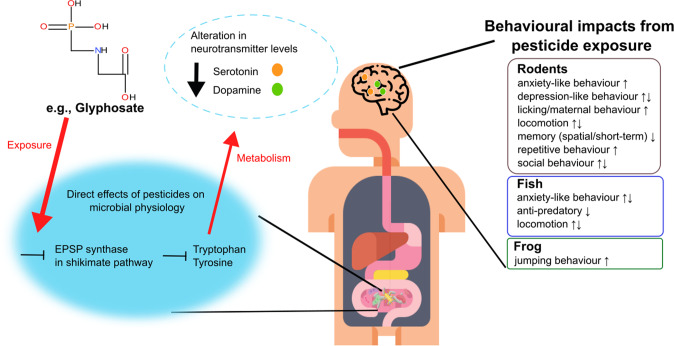
Effects of pesticides on animal behaviour via the microbiota-gut-brain axis. For example, for glyphosate, the mode of action is the inhibition of 5-enolpyruvylshikimate-3-phosphate (EPSP) synthase involved in the shikimate pathway. This may, in turn, alter the levels of final products of the pathway such as phenylalanine, tryptophan and tyrosine in bacteria. These aromatic amino acids are precursors for neurotransmitters used for brain communication, which could explain the behavioural impacts found upon exposure to glyphosate and other pesticides. Listed on the right are behavioural changes, such as anxiety, depression, memory and locomotor activity, found upon pesticide exposure in rodents, fish and frogs. The icons used in this figure were designed by Flat icons and Freepik on https://www.flaticon.com/ and the chemical structure was drawn using BIOVIA, Dassault Systèmes, BIOVIA Draw 2022, San Diego: Dassault Systèmes, 2023.

Behavioural readouts in animal studies are one of the underexploited outcome measures when assessing the impact of pesticides, especially when consideringthe impact on the gut microbiota (Supplementary Tables 1-3). Despite the limited number of studies conducted, pesticide exposure has been shown to impact host behaviours such as anxiety [43], memory [44] and social interaction [45] (Fig. 2). The mechanisms underpinning these pesticide-associated behavioural alterations remain to be conclusively verified, although a range of targets have been evaluated. It remains to be fully determined whether these changes are due to physiological, chemical, genetic, a combination of these and/or some other reasons. Thus, an interesting question for microbiologists is: can changes in gut bacteria structure and function drive these adverse effects on host behaviour? Changes in brain development and function are plausible consequences of disrupted signalling along the microbiota-gut-brain axis, considering the potential communicational alterations caused by the gut microbiota compositional and functional alterations. It has been previously proposed that gut microbial changes can induce behavioural impairments in the host by several mechanisms including disruptions in endocrine (e.g., HPA axis), neuronal and immune pathways [46]. Although hypothetical, it is worth exploring whether pesticide-driven activation of the HPA axis can trigger the immune system, resulting in a modification of microbial diversity, which would likely be detrimental to gut function. This change in the intestinal microbial composition and structure could result in altered production of various metabolic biproducts, which can in turn stimulate both the enteric nervous system and the vagal afferent nerves and contribute to additional activation of the HPA axis. However, this would require more evidence prior to elucidating facts.

### Glyphosate—the prototypical herbicide

N-(phosphonomethyl) glycine, commonly known as glyphosate, is the active compound in several widely used formulations referred to as glyphosate-based herbicides, for example, Roundup and it has been licensed for use in the EU since 2002 with its approval set to expire in December 2023 (European Food Safety Authority). These formulations function as an herbicide primarily by inhibiting the enzyme 5-enolpyruvylshikimate-3-phosphate synthase (EPSPS) involved in the shikimate pathway, which takes part in the production of aromatic amino acids [33]. These aromatic amino acids, phenylalanine, tyrosine and tryptophan, assist protein synthesis in plants. The glyphosate-based herbicides have been claimed to be low risk for human health due to a lack of the shikimate pathway in mammals [33]. However, this idea is being unravelled as faulty as more research is recording negative impacts of glyphosate on non-target organisms that possess the shikimate pathway, such as microbial communities residing in the gut [47] (Fig. 2). It is worth noting that the aforementioned amino acids produced by this pathway are precursors for neurotransmitters, which are known to play an important role in mood, behaviour and cognition. Indeed, dose-dependent reduction of serotonin, dopamine and norepinephrine, was confirmed in the brain of rats exposed to glyphosate [48].

Several studies have confirmed that exposure to glyphosate, either in its pure chemical form or in an herbicide mixture, has been shown to induce changes in host behaviour (Supplementary Table 1). For example, 250 and 500 mg/kg/day of glyphosate exposure via Roundup to mice led to a reduction in locomotor activity [49], which is linked to complex human activities including motivation and learning. More relevantly, the exposure to glyphosate was found to increase anxiety and depressive-like behaviour while decreasing social interaction [49–51]. This could be due to a reduction in serotonin immunoreactive neurons in the dorsal raphe nucleus, basolateral amygdala and ventral medial prefrontal cortex upon pesticide exposure, which further indicates a disruption in mood-regulating neurotransmitters (e.g. serotonin) in mice [49]. Alternatively, hyperactivation recorded in medial prefrontal cortex and amygdala regions [52], which are known to be involved in emotional processes including fear circuits [53] and may also explain the behavioural impairments. However, the impact on anxiety and depressive-like behaviour was diminished when the exposure was reduced to an environmentally relevant dosage [54]. While this discrepancy could be due to differences in behavioural tests applied, it highlights the need for replication of the study with a lower dose.

In rats, maternal licking behaviour was increased upon Roundup exposure, but not glyphosate [20]. This change in maternal behaviour could extend to emotional dysregulation in the offspring, though the measurement was not included in the study. Moreover, only licking behaviour and not the other maternal behaviours tested were affected, making it difficult to conclude the consequences of exposure to the pesticide. Currently, the explanation for glyphosate exposure and behavioural changes are based on exposure studies, which restricts our elucidation of any underlying causational mechanisms. These behavioural changes observed may also be explained by changes in the central nervous system, such as altered level of phagocytic cells in the cortical brain tissue [54], genetic expression of tight-junction genes [55] as well as the maturation level of doublecortin-immunoreactive neurones in the dorsal dentate gyrus of the hippocampus [20]. More recently, it was found that glyphosate enters the brain and elevates pro-inflammatory cytokines [56]. Increased levels of pro-inflammatory cytokines are commonly observed after traumatic brain injury, inducing both beneficial and detrimental effects. For example, tumour necrosis factor (TNF) has been shown to both increase neuronal cell death and promote neuroprotection in a receptor-type dependent manner [57]. Notably, increased TNF is a known characteristic of Alzheimer’s disease [58]. Thus, increased TNF in brain plasma, hippocampus and cortex may be indicating neurotoxic effects from glyphosate exposure [56].

Incidentally, an increase in *Bacteroidota* and decrease in *Bacillota* phyla within the gut were generally consistent observations in rats exposed to glyphosate (Supplementary Table 1). This trend was consistent despite the variation in the experimental design (e.g., ways of administration, exposure duration, microbiome analysis technique) [20, 21, 59, 60]. Overall, together with behavioural changes upon glyphosate exposure, significant changes in the proportion of some bacterial taxa within the gut microbiota were observed. Both affected phyla can produce neuromodulators including neurotransmitters, the metabolites of their precursors, SCFAs and bile acids [61], which could be linked with the behavioural impairments observed. Pre- and postnatal maternal glyphosate exposure in mice increased level of acetic acid in the offspring’s faecal samples according to metabolomic analysis [51]. Indeed, it has been shown that the profile of amino acid biosynthesis can be predicted from the host gut microbiome status [62], which suggests that biologically available neuromodulators are altered due to microbiome alterations from pesticide exposure. All of these findings point to the chemical impact on the microbiota-gut-brain axis having multiple underlying mechanisms.

### Pesticides altering physical and biochemical characteristics of the gut microbiota environment

Studies using other pesticides further strengthen the point that altered signalling to the brain by modified gut-microbiota-produced neuromodulators could explain behavioural impairments. Thymol is a plant extract based fungicide, which is suggested to have beneficial impacts such as immunomodulatory and anti-inflammatory functions [63]. While several modes of action are being proposed, with a primary target being cell membrane disruption, the whole picture has yet to be resolved. It has also been shown that thymol may bind to GABA receptors, which may interfere with signalling within the nervous system [64]. In fact, when zebrafish larvae were exposed to thymol, for 96 hours, fear and anxiety-like behaviours were disrupted as measured by reduced response to threat and increased distance moved during the dark phase. However, no change in exploratory behaviour was observed [65]. It should be noted that this study had a low sample size (*n* = 5/treatment), thus requiring a further replication to confirm this finding. While this study did not include microbiota analysis per se, when thymol-mixture exposed microbiota were introduced to germ-free zebrafish, their immune response was altered indicating a causational impact of pesticide exposure mediated by microbial changes [66]. Future studies focusing on the effect of pure thymol exposure will allow us to understand the direct effect of the given pesticide. In addition, exposure to the fungicide propamocarb, which similarly to thymol targets disruption of the cell membrane, increased propionate, isobutyrate and bile acids in faeces of exposed mice according to a faecal metabolomics analysis. This could partially be explained by a change in levels of SCFA producing bacteria such as *Ruminococcus, Bacteroides* and *Oscillospira* [67]. However, the low explained variation in the principal coordinates analysis, demands stronger evidence, which could confirm this treatment dependent microbiota changes. Taken together, it may be speculated that thymol exposure led to changes in the gut-microbiota-derived neuromodulators impacting the host behaviours. Other studies observed that exposure to the herbicide glufosinate ammonium led to behavioural impairments in locomotor activity, social and memory tests in mice (Supplementary Table 1). Glufosinate ammonium interferes with glutamate-glutamine homeostasis, which are main excitatory and inhibitory neurotransmitters, essential for neuronal activity. One of the studies also confirmed changes in the gut microbiota, including a decrease in the proportion of *Bacillota*. In this semi-longitudinal study (4, 6 and 8 weeks), microbiome analysis (16 S gene amplicon sequencing) and metabolomics (pathway enrichment) analysis on faecal samples highlighted retinol metabolism and fatty acid biosynthesis as one of the pathways altered by glufosinate ammonium exposure [68].This again illustrates that changes in amino acid and vitamin production can further harm the symbiotic relationship between the microbes in the gut and host by altering the gut microbiome community [69, 70]. They have further presented strong evidence of the causal relationship between gut microbiota and some social behavioural impairments by conducting faecal microbiota transplantation (FMT) [68]. Altogether, it further strengthens that glufosinate ammonium induced shifts in microbiota community structure, which can alter the biochemical environment (e.g., retinol), in the gut.

In another study in mice, chronic exposure (10 weeks) of glufosinate ammonium increased hippocampal glutamine synthetase activity [71], which may be due to the pesticides mode of action of competitive and irreversible inhibitor of the given enzyme. As glutamine synthetase assists in glutamate homeostasis, this disruption in the hippocampal activity level could explain the memory impairments also recorded in the same study. Coincidentally, rats exposed to an insecticide permethrin, which also interferes with nervous system signalling by modulating sodium channels, showed disturbed memory consolidation as well as altered hippocampal morphology. Morphological changes included reduction in the number of synapses, synaptic surface densities and ratio of perforated synapses in hippocampal areas, such as stratum moleculare of CA1, mossy fibres and inner molecular layer of the dentate gyrus [72].

Changes in the brain were also observed when rats were exposed to penconazole, a fungicide which functions by inhibiting the demethylation of sterol biosynthesis. A study showed changes in cerebrum, such as neuronal degeneration, neurophagias and cerebellum, including cell necrosis. While these biological consequences in animals are understudied, the exposed rats also presented increased depressive- and anxiety-like behaviours and decreased spatial memory after 10 days of exposure. However, unlike glufosinate ammonium, no histopathological changes were observed in the hippocampus despite its importance for memory [43]. It will be interesting to see if replication of this study verifies and extends the original observations towards a more detailed mechanistic understanding. While this study did not analyse gut microbiota changes, it has been shown that penconazole alters its composition in mice exposed to the chemical for four weeks [73]. Although the authors did not draw any direct conclusions on the mechanism, the Pearson correlation analysis of gut microbiota and metabolic profile highlighted that the perturbations in the gut microbiome may impact lipid and glucose metabolism through numerous pathways including an interference with bile acid secretion.

Brain changes by pesticides are not limited to its morphology but also to its metabolism. For instance, gut microbiome, brain and faecal metabolome were impacted when mice were exposed to aldicarb, an insecticide which works by inhibiting acetylcholinesterase activity, for 13 weeks. Accumulating evidence supports the microbiome being the key player in brain metabolism, by analysing the faeces, as seen from metagenomic analysis showing increase in bacterial enzymes involved in protein degradation as well as metabolomic analysis presenting critical brain metabolites including glucose, malic acid and free fatty acids being reduced, while a ketone body was increased. These changes together support that brain energy metabolism is being negatively affected by pesticide exposure [74]. Overall, these findings suggest gut microbiota metabolites altered by pesticide exposure may induce general behavioural impairments on the host via the microbiota-gut-brain axis, while some impacts may be by pesticide-specific processes. However, causational studies are strongly warranted to understand the specific involvement of gut microbiota in the process.

### Pesticides impacting on gut and brain barriers of the microbiota-gut-brain axis

Another mechanism to explain the behavioural impairments observed upon pesticide exposure could be due to disruption in barrier function, thus altering the permeability of gut and brain. The gut microbiota is known to have a key role in preventing damage to the intestinal barrier [75]. Therefore, it is possible that gut microbiota change via pesticide exposure is having a downstream effect by impacting barrier function. Belonging to neonicotinoids group, imidacloprid is an insecticide that targets nicotinic acetylcholine receptors. Studies have shown that exposure to imidacloprid can lead to impaired behaviour in rodents. Indeed, a decrease in locomotor activity and an increase in stress-like behaviours were recorded in adult mice exposed to imidacloprid for 60 days. This stress response was also confirmed via biochemical changes of related hormones [76]. On the contrary, there is strong evidence of an increase in locomotor activity and a reduction in depressive-like behaviours in rats exposed to the same chemical for 25 days [77]. This divergence may be explained by the difference in animal models, exposure duration or exposure time window (directly in adulthood vs indirectly via the mother during early life). However, it requires additional studies to draw firmer conclusions.

While these behavioural studies did not assess the gut microbiota, separate mice studies found that imidacloprid affects the proportion of *Bacillota* and *Verrucomicrobiota* within the gut microbiota [78, 79]. It was also observed that imidacloprid exposure disrupted bile acid metabolism and colon barrier function, which was confirmed by changes in expression of related genes such as *cyp7a1, fgf15, zo-1* and *claudin-1*. This reduction in gram-negative bacteria (e.g., *Akkermansia*), an increase in gram-positive bacteria (e.g., *Allobaculum*) and disruption in colon barrier function was also confirmed by gene expression analysis [79]. Furthermore, the exposure to insecticide chlorpyrifos in rats, another pesticide targeting the acetylcholine cycle by inhibiting acetylcholinesterase activity, led to changes in the gut microbiota profile (both by traditional culture method and gene expression analysis) and increased intestinal permeability (via fluorescein isothiocyanate (FITC)–dextran trans-epithelial permeability assay) as well as bacterial translocation to the spleen [80, 81]. However, behavioural analysis is currently lacking for hosts exposed to chlorpyrifos, thus requiring further experimental evidence.

In contrast to the gut, the brain is protected by the highly selective blood brain barrier (BBB) in order to closely regulate what enters the central nervous system [82]. Microbially derived SCFAs are crucial in microbiota-gut-brain axis signalling, including in the maintenance of BBB integrity [83, 84]. Furthermore, administration of various insecticides to rats (cypermethrin, lindane and quinalphos) have been shown to impair BBB permeability, especially in early life [41]. All of these insecticides interfere with neuronal signalling while having different targets: sodium channel, GABA-gated chloride channel and acetylcholinesterase, respectively. Coincidentally, neonatal exposure to another pyrethroid pesticide, permethrin, has been shown to increase locomotor activity and reduce memory in adulthood compared to vehicle treated rats across replicated cages. Permethrin exposure also reduced synapse and surface densities in brain regions such as dentate gyrus and hippocampus, potentially explaining the behavioural impairments observed [72]. The research group continued to analyse the faecal microbiota with the same experimental setup and found that the proportion of *Bacteroides*, *Prevotella* and *Porphyromonas* were reduced, while *Enterobacteriaceae* and *Lactobacillus* were increased. However, it is important to note that changes in some bacterial groups may have been overlooked, due to the reliance on the Real-Time quantitative PCR based analysis. Additionally, changes in bacterial faecal metabolites such as acetic and propionic but not butyric acid, were detected upon permethrin exposure [85]. These observations together strengthen the argument that pesticide exposure and altered microbially derived SCFA levels leading to behavioural changes, potentially by disturbing integrity and the function of the physical barriers of the brain and the gut.

### Pesticides influencing the immune system

The status of the immune system can have an impact on host behaviours, which can be influenced by changes within the gut microbiota. For example, impacts on microglia cells, the most dominant immune cells in the brain, can lead to disruption in synaptic pruning, myelination regulation, and neurogenesis [86]. Microglial immune cells are activated by an infection by microbial pathogens with signalling from lipopolysaccharide, a pro-inflammatory molecule which reside in outer membrane component of gram-negative bacteria [87]. Indeed, when mice were exposed to chlorpyrifos, levels of lipopolysaccharide increased [88] indicating that the pesticide induced an immune response. This immune activation of microglia can cause a signalling cascade to molecules such as the transcription factor, NF-κB, which amplifies the immune and inflammatory response [89]. In the brain, NF-κB also interfere with glial and neuronal cell function, potentially worsening diseases such as Alzheimer’s [90, 91]. These pro-inflammatory cytokines produced as an immune response are known to be related to symptoms such as anxiety and depression both in animals and humans [92, 93].

The potential of pesticides to exert an impact via the immune system is also supported, for example, in studies using atrazine. Atrazine is an herbicide which interferes with photosynthesis in plants, but is also known to disrupt the endocrine system in amphibians and mammals. When frogs were exposed to atrazine, it led to a reduction in gut microbiota diversity as well as a significant increase in the proportion of *Lactobacillus* and *Weisella* for the highest dosage tested (500 μg/L). In addition to changes in gut microbiota, there were altered behaviours of increased jumping distance and time [94]. Unfortunately, this study did not analyse its impact on immunity. However, transcriptomics and proteomics revealed the downregulation of gene expression and proteins related to immunity when wasps were exposed to the same pesticide [95]. Gene expression via both qualitative polymerase chain reaction and RNA-sequencing give further insights into the potential impacts of pesticide exposure on immune-related functions regardless of pesticide or animal model studied (Supplementary Tables 1-2) [96–98]. Taken together, these studies suggest that gut bacteria compositional changes exert immunomodulatory effects that could plausibly impact host behaviour. However, there is yet to be a study which included both immune functions and behavioural readouts. It is strongly suggested that including behaviour with gut microbiota studies are required to understand the significance of disrupted microbiota-gut-brain axis signalling upon pesticide exposure.

Not all pesticide exposure studies have found behavioural impairments on the animals, suggesting that these effects may not simply be solely pesticide dependent. For example, several studies using glufosinate ammonium failed to observe a significant impact on anxiety levels [45, 68, 71]. These conflicting findings warrant the need to uncover the mechanistic processes involved. While several mechanisms can be speculated, more studies including both gut microbiota and behavioural changes are required to assess whether recorded behavioural changes are happening via gut bacterial interactions with neuromodulators, neural pathways and immune system, in combination, or by other means. When interpreting behavioural readouts in preclinical models, consideration of the associated limitations should not be neglected [4]. In order to validate the results from preclinical models, studies incorporating other measures to assess CNS and cognitive processes, such as neuroimaging warrant further investigation. Animal models have been widely utilised for studying microbiota-brain interactions and its mechanisms [99]. By taking a holistic approach across species, this preclinical step will facilitate future translational studies for applying to human health [9].

### Pesticide induced changes to gut microbiota composition is dependent on host species

The implications of pesticide exposure on the gut microbiota have been explored more thoroughly and differently, compared to behavioural outcomes. This can be seen from the number of studies aimed at understanding this relationship using various animal models including mammals (mice, rats), insects (flies, beetles) amphibians (frogs), fish (carp, zebrafish) and other aquatic species (e.g., oyster) (Supplementary Tables 1-3). The general consensus suggests that pesticide exposure alters the gut microbiota, albeit the differences in the affected microbial groups and its directionality. This variation may partially be explained by the difference in experimental design such as chemical type and dosage/duration (Supplementary Tables 1-3). However, it seems that experimental design is not the only reason for this divergence in microbial signatures post pesticide exposure. Despite this dissimilarity of findings, several patterns are worth noting. Foremost, for all chemical types, the majority of the impacted bacterial groups belong to either *Actinomycetota*, *Bacillota*, *Bacteroidota* and *Pseudomonadota* phylum (Supplementary Tables 1-3). In addition to this, there were some species-dependent trends in gut microbiota profile alterations, specifically in honeybees and mammals.

### Key microbial taxa impacted by pesticide exposure in honeybees

The blight of honeybee populations has been widely documented and reported. Although direct physiological impacts to the host are undoubtedly relevant, alterations to key microbial taxa through pesticide exposure have been shown with the specialised nature of the bee gut microbiota making them more susceptible to subsequent infection. Two key microbial groups belonging to the *Pseudomonadota* phylum, *Snodgrassella alvi* and *Gilliamella apicola* have been shown to generally decrease when honeybees were exposed to glyphosate [19, 100–102]. *Gilliamella* sp. were also found to be negatively affected by exposure to the insecticide nitenpyram, an insecticide which acts as a competitive modulator of nicotinic acetylcholine receptor, interfering with neuromascular functions [98]. It is known that *S. alvi* plays a key role in bee health by regulating the hosts immune response [103]. This decrease in immune levels via glyphosate exposure was confirmed where bee populations were found to be more susceptible to infection by opportunistic bacterial pathogens, for example by *Serratia marcescens* [100, 101], with this bacterial group also being found to increase after atrazine exposure in wasps [95]. The fact that these bacterial groups were not found to be changed in any other animal models may indicate that there could be species-pesticide specific interactions. Moreover, alterations in bee gut microbiota have also been recorded in larvae and immature bees. For example, a high dose of glyphosate (20 mg/L) has been shown to result in a distinctive midgut bacterial profile compared to lower dose (0.8 and 4 mg/L) and control [104]. However, results from an environmentally relevant dosage are yet to be fully understood.

### Key microbial taxa impacted by pesticide exposure in mammals

The reduction in the abundance of bacterial groups within *Bacillota* is a general trend found in animal studies using mammalian models, particularly mice and rats, when exposed to herbicides including but not limited to glyphosate (Supplementary Tables 1-3) [20, 21, 50, 60, 68, 105], fungicides [73, 106, 107] as well as insecticides [108–110]. The overall reduction in *Bacillota* phylum could be explained by a significant reduction of *Lactobacillaceae*/*Lactobacillus*. *Lactobacillus* is a known beneficial bacterial genus with many members associated with functions such as enhancing intestinal barrier, for example, by increasing gene expression related to tight junction signalling [111]. The probiotic characteristics of some *Lactobacillus* strains also extend to involvement in modification of immune signalling between dendritic cell and T cells [112] as well as production of SCFAs and neurotransmitters [113]. Some *Lactobacillus* sp. can also protect intestinal barrier functions and help maintain the mucus layer, which were both found to be compromised after the fungicide imazalil was exposed in C57Bl/6 mice [114]. Imazalil works as a fungicide by inhibiting demethylation required for ergo/sterol biosynthesis. Concurrently, 16 S RNA gene amplicon sequencing showed that *Lactobacillus* was also negatively impacted by this exposure, suggesting a role here in intestinal homeostasis. Conversely, an increase in some genus of *Bacillota* was shown in mice upon exposure to propamocarb, which indicates a distinct pesticide-bacteria interaction for certain chemicals [67, 115, 116]. In relation to behaviour, administering *L. rhamnosus* has resulted in reduced anxiety- and depression- related behaviour in mice [117, 118], together with altered mRNA expression of GABA receptors in several brain regions [118]. Therefore, reduction in this beneficial bacterial group from pesticide exposure may be one potential factor of its detrimental impact on host behaviour. A major environmental concern is that this type of impact may be translated to humans.

On the contrary, *Lactobacillus* has been shown to generally increase in relative abundance when insects (honeybee, fly and drosophila) were exposed to pesticides (Supplementary Tables 1-2) [19, 119–121]. These results highlight once again that pesticide exposure can differentially impact specific genera of the gut microbiota in a host-species specific manner. However, the potential that these taxa-specific changes are achieved through the direct action of the pesticide on the microbes or through modulation of host metabolism remains unclear.

### Pesticides and the microbiome: need for functional analysis

Few studies have examined the direct functional implications of microbiome changes via relevant molecular approaches. This is surprising given the insights that functional-based molecular approaches can provide. Indeed, faecal and caecal metabolomics have revealed pesticide-mediated impacts on various functions including detoxification [119], amino acid metabolism [122], intestinal barrier function [123] and inflammation [124]. However, it is important to note that amplicon and shotgun sequencing based pathway analysis is not sufficient to conclude that the pesticide is the direct driver of these changes. This is why incorporating functional analysis is crucial as it allows us to clarify the implications of the gut microbiome community-wide alterations and examine direct impact(s), which may contradict the known function of a specific bacterial group. The tolerance and susceptibility of the gut ecosystem against pesticides was hinted to be also linked to life stages [125], sex [60, 105, 126], diet [127] and presence of pesticide-degrading strains [95]. Microbial ecosystems are complex and exhibit fascinating characteristics, such as functional redundancy, indicating that despite differences in composition, the overall function may not be impacted. Due to evolutionary conservation of functional microbial metabolite classes, a cross-species approach to investigate pesticide effects on microbiota-brain axis, may also shed new mechanistic insightst [99]. Therefore, future studies should incorporate 1) studies targeting the mechanistic and biological interactions between the pesticides and bacterial groups and 2) the functional implications of the microbiome ecosystem-wide changes.

## Future directions and conclusions

Due to the ecological nature of functional redundancy, the impacts of change in the gut microbial ecosystem as a whole becomes extremely important. One limitation of the current paradigms in pesticide exposure and the gut-brain axis is the lack of definitive studies that established causational relationships. One way to causally examine this is by conducting FMT: a powerful method in rodent and other animal models, which can assess whether the behavioural changes observed was induced by host-microbiota interactions, by administering the faeces from a host to donor to reproduce the host microbiota in the donor’s gut [128, 129]. FMT studies have been applied in a range of studies and have shown that gut microbiota profile plays an important role in determining the host’s resistance towards pesticides as seen in cockroaches and suggested for rodents [130, 131]. An exciting study also showed that some of the behavioural impairments observed by glufosinate ammonium exposure were transferrable by transplanting the gut ecosystem [68]. More studies incorporating pesticide-altered gut microbiota transplantation will allow us to investigate the impacts of gut ecosystem changes in driving behavioural impairments.

It is evident that numerous studies have been conducted to assess the impact of pesticides on the gut microbiota and/or behaviour (Supplementary Tables 1-3). Moving forward, it is crucial to learn and extend from the experimental designs and data already presentated in this field. When designing preclinical studies using animal models, replicating the dosage, duration and ways of administration from previous studies will provide firmer evidence on the findings. Especially for the dosage, as from a microbiota-gut-brain axis point of view, the interest lies in the environmentally relevant dosage, which is not a common dosage tested as toxicology-geared studies but will provide significantly higher societal value. Moreover, some of the studies masked its quality of support for interpretation as they did not incorporate, or mention, independent cage replication. Housing conditions, such as whether an animal was individually housed or group housed, can have consequential effects on animal behaviours [132]. It is also important to pay careful consideration to the statistical approach used to analyse microbiome data. As the ways to approach microbiome data are always evolving, establishing a standard microbiome analysis protocol is challenging. Therefore, the responsibility lies on the researchers to provide precise details on how the analysis was conducted to accommodate reproducibility of findings [133]. When considering the relative abundance of a given taxa, the analysis applied should be finely tuned depending on the research question [134]. All this information is crucial to be included in the manuscript for future studies to be replicated accordingly.

Another crucial direction within this field is to have a clearer understanding of the direct interaction between pesticides and the microbiota. It is not understood whether the tolerance/susceptibility against pesticides are due to the ecosystem as a whole or due to a bacterial taxa group level difference [95], while the answer to this is being revealed by using systemic approaches [135] and in vitro models, such as Simulator of the Human Intestinal Microbial Ecosystem (SHIME) [108, 136]. More research needs to be targeted to understand what is driving the divergent impacts on gut microbiota upon pesticide exposure. It would also be important to incorporate confounding factors and to examine the relevance of changes in the functional context. While studies suggest a role of microbiota-gut-brain axis as the underlying mechanism for driving adverse effects on behaviours, further studies are required to assess other potential reasons such as genetic and physiological influences. By adding the understanding of microbiota-driven impact of pesticides, to its already known direct impact on host biology [124], we will be able to understand the full picture of the complex interaction between the pesticide and host.

**Fig. 3:**
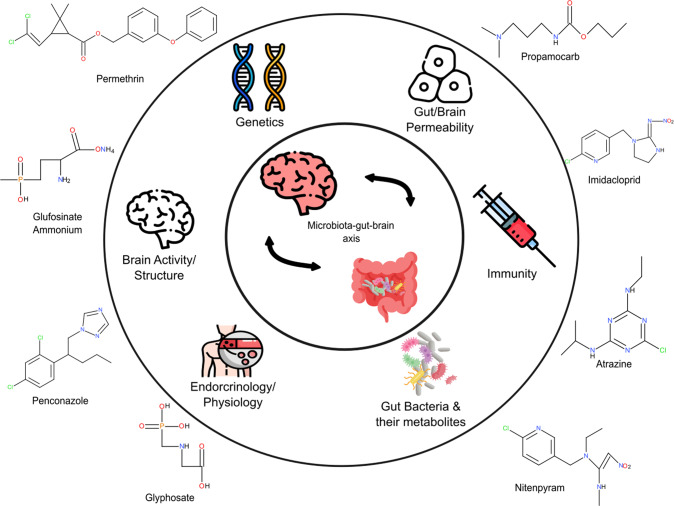
Potential mediators between pesticides influencing animal behaviour. The mechanism(s) recruited following pesticide exposure that lead to impaired behaviours remains to be defined. It is possible that behavioural impairments are being caused by changes in endocrinology/physiology, brain activity/structure, genetics, gut/brain permeability, immunity or gut bacteria and their metabolites. Further investigations are warranted to clarify whether one of these components, or combinations thereof, are influencing the microbiota-gut-brain axis. The icons used in this figure were designed by Flat icons, Freepik and surang on https://www.flaticon.com/ and the chemical structure was drawn using BIOVIA, Dassault Systèmes, BIOVIA Draw 2022, San Diego: Dassault Systèmes, 2023.

While growing attention rests on the potential risk of pesticide usage, the impact on the microbiota-gut-brain axis seems to be a neglected aspect of research in the field. The negative impact pesticides have on the host behaviour may be exerted through an altered gut ecosystem, which may potentially be a crucial biomarker reservoir for examining chemical toxicity. As presented in this review, a substantial number of findings collectively indicate that pesticide exposure during the lifetime and before birth leads to alterations in gut microbiota profiles and host behaviours, such as anxiety/depressive-like behaviours and memory. However, studies that have directly investigated whether the behavioural impairments are being mediated by the gut microbiota are limited (Fig. 3). It is also worth noting that this review did not focus on other notable factors such as 1) all the other active ingredients available on the market, 2) synergistic effects of pesticide mixtures, 3) morphological/histological differences and 4) other environmental factors that could also pose a threat to gut microbiota, such as heavy metals and industrial by-products. Most importantly, unravelling the relationship between pesticides, microbiota and brain (behaviour), will lead to essential discussions on sustainable ways to cohabitate with “safe” pesticides to conserve the ecosystem and health of existing organisms.

## Supplementary information

Supplementary material

Supplementary References

## Data Availability

Data sharing not applicable to this article as no datasets were generated or analysed during the current study.
