# Supplementary material for: Pesticide exposure and the microbiota-gut-brain axis
Source: ISME J. 2023 Jun 16;17(8):1153–66. doi: 10.1038/s41396-023-01450-9 (PMC10356795; doi:10.1038/s41396-023-01450-9)
Supplement: Supplementary file 2 — Supplementary References [file 41396_2023_1450_MOESM2_ESM.docx]

**Supplementary References**

1. Tu P, Gao B, Chi L, Lai Y, Bian X, Ru H, et al. Subchronic low-dose 2,4-D exposure changed plasma acylcarnitine levels and induced gut microbiome perturbations in mice. *Sci Rep Nat Publ Group* 2019; **9**: 4363.

2. Pompermaier A, Varela ACC, Mozzato MT, Soares SM, Fortuna M, Alves C, et al. Impaired initial development and behavior in zebrafish exposed to environmentally relevant concentrations of widely used pesticides. *Comp Biochem Physiol Part C Toxicol Pharmacol* 2022; **257**: 109328.

3. Brown JB, Langley SA, Snijders AM, Wan KH, Morris SNS, Booth BW, et al. An integrated host-microbiome response to atrazine exposure mediates toxicity in *Drosophila*. *Commun Biol* 2021; **4**: 1324.

4. Knutie SA, Gabor CR, Kohl KD, Rohr JR. Do host-associated gut microbiota mediate the effect of an herbicide on disease risk in frogs? *J Anim Ecol* 2018; **87**: 489–499.

5. Zhao Q, Huang M, Liu Y, Wan Y, Duan R, Wu L. Effects of atrazine short-term exposure on jumping ability and intestinal microbiota diversity in male *Pelophylax nigromaculatus* adults. *Environ Sci Pollut Res* 2021; **28**: 36122–36132.

6. Liu B, Zeng Q, Chen H, Liao J, Bai Y, Han Q, et al. The hepatotoxicity of altrazine exposure in mice involves the intestinal microbiota. *Chemosphere* 2021; **272**: 129572.

7. Britt A, Bernini M, McSweeney B, Dalapati S, Duchin S, Cavanna K, et al. The effects of atrazine on the microbiome of the eastern oyster: *Crassostrea virginica*. *Sci Rep* 2020; **10**: 11088.

8. Wang G-H, Berdy BM, Velasquez O, Jovanovic N, Alkhalifa S, Minbiole KPC, et al. Changes in microbiome confer multigenerational host resistance after sub-toxic pesticide exposure. *Cell Host Microbe* 2020; **27**: 213–224.

9. Simonsen D, Cady N, Zhang C, Shrode RL, McCormick ML, Spitz DR, et al. The effects of benoxacor on the liver and gut microbiome of C57BL/6 mice. *Toxicol Sci Off J Soc Toxicol* 2022; **186**: 102–117.

10. Castelli L, Branchiccela B, Zunino P, Antúnez K. Insights into the effects of sublethal doses of pesticides glufosinate-ammonium and sulfoxaflor on honey bee health. *Sci Total Environ* 2023; **868**: 161331.

11. Calas A-G, Richard O, Même S, Beloeil J-C, Doan B-T, Gefflaut T, et al. Chronic exposure to glufosinate-ammonium induces spatial memory impairments, hippocampal MRI modifications and glutamine synthetase activation in mice. *NeuroToxicology* 2008; **29**: 740–747.

12. Dong T, Guan Q, Hu W, Zhang M, Zhang Y, Chen M, et al. Prenatal exposure to glufosinate ammonium disturbs gut microbiome and induces behavioral abnormalities in mice. *J Hazard Mater* 2020; **389**: 122152.

13. Laugeray A, Herzine A, Perche O, Hébert B, Aguillon-Naury M, Richard O, et al. Pre- and postnatal exposure to low dose glufosinate ammonium induces autism-like phenotypes in mice. *Front Behav Neurosci* 2014; **8**: 390.

14. Gómez-Gallego C, Rainio MJ, Collado MC, Mantziari A, Salminen S, Saikkonen K, et al. Glyphosate-based herbicide affects the composition of microbes associated with Colorado potato beetle (*Leptinotarsa decemlineata*). *FEMS Microbiol Lett* 2020; **367**: fnaa050.

15. Chen J, Rao C, Yuan R, Sun D, Guo S, Li L, et al. Long-term exposure to polyethylene microplastics and glyphosate interferes with the behavior, intestinal microbial homeostasis, and metabolites of the common carp (*Cyprinus carpio L.*). *Sci Total Environ* 2022; **814**: 152681.

16. Yan B, Han J, Sun Y, Lei L, Yuan J, Qiao Z, et al. Probiotics ameliorate growth retardation of glyphosate by regulating intestinal microbiota and metabolites in crucian carp (*Carassius auratus*). *Sci Total Environ* 2022; **851**: 158260.

17. Yang X, Song Y, Zhang C, Pang Y, Song X, Wu M, et al. Effects of the glyphosate-based herbicide roundup on the survival, immune response, digestive activities and gut microbiota of the Chinese mitten crab, Eriocheir sinensis. *Aquat Toxicol* 2019; **214**: 105243.

18. Cullen MG, Bliss L, Stanley DA, Carolan JC. Investigating the effects of glyphosate on the bumblebee proteome and microbiota. *Sci Total Environ* 2023; **864**: 161074.

19. Blot N, Veillat L, Rouzé R, Delatte H. Glyphosate, but not its metabolite AMPA, alters the honeybee gut microbiota. *PLoS One* 2019; **14**: e0215466.

20. Motta EVS, Moran NA. Impact of glyphosate on the honey bee gut microbiota: effects of intensity, duration, and timing of exposure. *mSystems* 2020; **5**: e00268-20.

21. Motta EVS, Raymann K, Moran NA. Glyphosate perturbs the gut microbiota of honey bees. *Proc Natl Acad Sci* 2018; **115**: 10305–10310.

22. Motta EVS, Mak M, De Jong TK, Powell JE, O’Donnell A, Suhr KJ, et al. Oral or topical exposure to glyphosate in herbicide formulation impacts the gut microbiota and survival rates of honey bees. *Appl Environ Microbiol* 2020; **86**: e01150-20.

23. Motta EVS, Powell JE, Moran NA. Glyphosate induces immune dysregulation in honey bees. *Anim Microbiome* 2022; **4**: 16.

24. Dai P, Yan Z, Ma S, Yang Y, Wang Q, Hou C, et al. The herbicide glyphosate negatively affects midgut bacterial communities and survival of honey bee during larvae reared in vitro. *J Agric Food Chem* 2018; **66**: 7786–7793.

25. Aitbali Y, Ba-Mhamed S, Bennis M. Behavioral and immunohistochemical study of the effects of subchronic and chronic exposure to glyphosate in mice. *Front Behav Neurosci* 2017; **11**: 146.

26. Aitbali Y, Ba-M’hamed S, Elhidar N, Nafis A, Soraa N, Bennis M. Glyphosate based- herbicide exposure affects gut microbiota, anxiety and depression-like behaviors in mice. *Neurotoxicol Teratol* 2018; **67**: 44–49.

27. Aitbali Y, Kaikai N, Ba-M’hamed S, Sassoè-Pognetto M, Giustetto M, Bennis M. Anxiety and gene expression enhancement in mice exposed to glyphosate-based herbicide. *Toxics* 2022; **10**: 226.

28. de Castro Vieira Carneiro CL, Chaves EMC, Neves KRT, Braga MDM, Assreuy AMS, de Moraes MEA, et al. Behavioral and neuroinflammatory changes caused by glyphosate: Base herbicide in mice offspring. *Birth Defects Res* 2022; **115**: 488–97.

29. Del Castilo I, Neumann AS, Lemos FS, De Bastiani MA, Oliveira FL, Zimmer ER, et al. Lifelong exposure to a low-dose of the glyphosate-based herbicide RoundUp® causes intestinal damage, gut dysbiosis, and behavioral changes in mice. *Int J Mol Sci* 2022; **23**: 5583.

30. Pu Y, Yang J, Chang L, Qu Y, Wang S, Zhang K, et al. Maternal glyphosate exposure causes autism-like behaviors in offspring through increased expression of soluble epoxide hydrolase. *Proc Natl Acad Sci* 2020; **117**: 11753–11759.

31. Iori S, Rovere GD, Ezzat L, Smits M, Ferraresso SS, Babbucci M, et al. The effects of glyphosate and AMPA on the mediterranean mussel *Mytilus galloprovincialis* and its microbiota. *Environ Res* 2020; **182**: 108984.

32. Ruuskanen S, Rainio MJ, Gómez-Gallego C, Selenius O, Salminen S, Collado MC, et al. Glyphosate-based herbicides influence antioxidants, reproductive hormones and gut microbiome but not reproduction: A long-term experiment in an avian model. *Environ Pollut* 2020; **266**: 115108.

33. Mesnage R, Panzacchi S, Bourne E, Mein CA, Perry MJ, Hu J, et al. Glyphosate and its formulations Roundup Bioflow and RangerPro alter bacterial and fungal community composition in the rat caecum microbiome. *Front Microbiol* 2022; **13**: 888853.

34. Dechartres J, Pawluski JL, Gueguen M-M, Jablaoui A, Maguin E, Rhimi M, et al. Glyphosate and glyphosate-based herbicide exposure during the peripartum period affects maternal brain plasticity, maternal behaviour and microbiome. *J Neuroendocrinol* 2019; **31**: e12731.

35. Liu J-B, Chen K, Li Z-F, Wang Z-Y, Wang L. Glyphosate-induced gut microbiota dysbiosis facilitates male reproductive toxicity in rats. *Sci Total Environ* 2022; **805**: 150368.

36. Lozano VL, Defarge N, Rocque L-M, Mesnage R, Hennequin D, Cassier R, et al. Sex-dependent impact of Roundup on the rat gut microbiome. *Toxicol Rep* 2018; **5**: 96–107.

37. Mao Q, Manservisi F, Panzacchi S, Mandrioli D, Menghetti I, Vornoli A, et al. The Ramazzini Institute 13-week pilot study on glyphosate and Roundup administered at human-equivalent dose to Sprague Dawley rats: effects on the microbiome. *Environ Health* 2018; **17**: 50.

38. Mesnage R, Teixeira M, Mandrioli D, Falcioni L, Ducarmon QR, Zwittink RD, et al. Use of shotgun metagenomics and metabolomics to evaluate the impact of glyphosate or Roundup MON 52276 on the gut microbiota and serum metabolome of Sprague-Dawley rats. *Environ Health Perspect* 2021; **129**: 017005.

39. Tang Q, Tang J, Ren X, Li C. Glyphosate exposure induces inflammatory responses in the small intestine and alters gut microbial composition in rats. *Environ Pollut* 2020; **261**: 114129.

40. Owagboriaye F, Mesnage R, Dedeke G, Adegboyega T, Aladesida A, Adeleke M, et al. Impacts of a glyphosate-based herbicide on the gut microbiome of three earthworm species (Alma millsoni, Eudrilus eugeniae and Libyodrilus violaceus): A pilot study. *Toxicol Rep* 2021; **8**: 753–758.

41. Ding W, Shangguan Y, Zhu Y, Sultan Y, Feng Y, Zhang B, et al. Negative impacts of microcystin-LR and glyphosate on zebrafish intestine: Linked with gut microbiota and microRNAs? *Environ Pollut* 2021; **286**: 117685.

42. Ivantsova E, Wengrovitz AS, Souders CL, Martyniuk CJ. Developmental and behavioral toxicity assessment of glyphosate and its main metabolite aminomethylphosphonic acid (AMPA) in zebrafish embryos/larvae. *Environ Toxicol Pharmacol* 2022; **93**: 103873.

43. Wang K, Zhang C, Zhang B, Li G, Shi G, Cai Q, et al. Gut dysfunction may be the source of pathological aggregation of alpha-synuclein in the central nervous system through Paraquat exposure in mice. *Ecotoxicol Environ Saf* 2022; **246**: 114152.

44. Li Y, Zuo Z, Zhang B, Luo H, Song B, Zhou Z, et al. Impacts of early-life paraquat exposure on gut microbiota and body weight in adult mice. *Chemosphere* 2022; **291**: 133135.

45. Qi M, Wang N, Xiao Y, Deng Y, Zha A, Tan B, et al. Ellagic acid ameliorates paraquat-induced liver injury associated with improved gut microbial profile. *Environ Pollut Barking Essex 1987* 2022; **293**: 118572.

46. Kan H, Zhao F, Zhang X-X, Ren H, Gao S. Correlations of gut microbial community shift with hepatic damage and growth inhibition of *Carassius auratus* induced by pentachlorophenol exposure. *Environ Sci Technol* 2015; **49**: 11894–11902.

47. Zeng F, Wu L, Ren X, Xu B, Cui S, Li M, et al. Effects of chronic prometryn exposure on antioxidative status, intestinal morphology, and microbiota in sea cucumber (*Apostichopus japonicus*). *Comp Biochem Physiol Part C Toxicol Pharmacol* 2021; **250**: 109187.

48. Santos KPE dos, Ferreira Silva I, Mano-Sousa BJ, Duarte-Almeida JM, Castro WV de, Azambuja Ribeiro RIM de, et al. Abamectin promotes behavior changes and liver injury in zebrafish. *Chemosphere* 2023; **311**: 136941.

49. Liu J, Zhao F, Wang T, Xu Y, Qiu J, Qian Y. Host metabolic disorders induced by alterations in intestinal flora under dietary pesticide exposure. *J Agric Food Chem* 2021; **69**: 6303–6317.

50. Gao B, Chi L, Tu P, Gao N, Lu K. The carbamate aldicarb altered the gut microbiome, metabolome, and lipidome of C57BL/6J mice. *Chem Res Toxicol* 2019; **32**: 67–79.

51. Kryukov VY, Rotskaya U, Yaroslavtseva O, Polenogova O, Kryukova N, Akhanaev Y, et al. Fungus *Metarhizium robertsii* and neurotoxic insecticide affect gut immunity and microbiota in Colorado potato beetles. *Sci Rep* 2021; **11**: 1299.

52. Qin D, Zheng Q, Zhang P, Lin S, Huang S, Cheng D, et al. Azadirachtin directly or indirectly affects the abundance of intestinal flora of Spodoptera litura and the energy conversion of intestinal contents mediates the energy balance of intestine-brain axis, and along with decreased expression CREB in the brain neurons. *Pestic Biochem Physiol* 2021; **173**: 104778.

53. Sun Z, Sun W, An J, Xu H, Liu Y, Yan C. Copper and chlorpyrifos stress affect the gut microbiota of chironomid larvae (*Propsilocerus akamusi*). *Ecotoxicol Environ Saf* 2022; **244**: 114027.

54. Sun Y, Pei J, Chen X, Lin M, Pan Y, Zhang Y, et al. The role of the gut microbiota in depressive-like behavior induced by chlorpyrifos in mice. *Ecotoxicol Environ Saf* 2023; **250**: 114470.

55. Chen AS, Liu DH, Hou HN, Yao JN, Xiao SC, Ma XR, et al. Dietary pattern interfered with the impacts of pesticide exposure by regulating the bioavailability and gut microbiota. *Sci Total Environ* 2023; **858**: 159936.

56. Zhao Y, Zhang Y, Wang G, Han R, Xie X. Effects of chlorpyrifos on the gut microbiome and urine metabolome in mouse (*Mus musculus*). *Chemosphere* 2016; **153**: 287–293.

57. Liang Y, Zhan J, Liu D, Luo M, Han J, Liu X, et al. Organophosphorus pesticide chlorpyrifos intake promotes obesity and insulin resistance through impacting gut and gut microbiota. *Microbiome* 2019; **7**: 19.

58. Fang B, Li JW, Zhang M, Ren FZ, Pang GF. Chronic chlorpyrifos exposure elicits diet-specific effects on metabolism and the gut microbiome in rats. *Food Chem Toxicol* 2018; **111**: 144–152.

59. Joly C, Gay-Quéheillard J, Léké A, Chardon K, Delanaud S, Bach V, et al. Impact of chronic exposure to low doses of chlorpyrifos on the intestinal microbiota in the Simulator of the Human Intestinal Microbial Ecosystem (SHIME®) and in the rat. *Environ Sci Pollut Res* 2013; **20**: 2726–2734.

60. Joly Condette C, Bach V, Mayeur C, Gay-Quéheillard J, Khorsi-Cauet H. Chlorpyrifos exposure during perinatal period affects intestinal microbiota associated with delay of maturation of digestive tract in rats. *J Pediatr Gastroenterol Nutr* 2015; **61**: 30–40.

61. Krishnaswamy VG, Jaffar MF, Sridharan R, Ganesh S, Kalidas S, Palanisamy V, et al. Effect of chlorpyrifos on the earthworm *Eudrilus euginae* and their gut microbiome by toxicological and metagenomic analysis. *World J Microbiol Biotechnol* 2021; **37**: 76.

62. Huang Z, Xiao X, Wang D, Zhong Y, Ding Q, You J. Joint effects of micro-sized polystyrene and chlorpyrifos on zebrafish based on multiple endpoints and gut microbial effects. *J Environ Sci* 2023; **126**: 184–197.

63. Wang X, Shen M, Zhou J, Jin Y. Chlorpyrifos disturbs hepatic metabolism associated with oxidative stress and gut microbiota dysbiosis in adult zebrafish. *Comp Biochem Physiol Part C Toxicol Pharmacol* 2019; **216**: 19–28.

64. Janssens L, Van de Maele M, Delnat V, Theys C, Mukherjee S, De Meester L, et al. Evolution of pesticide tolerance and associated changes in the microbiome in the water flea *Daphnia magna*. *Ecotoxicol Environ Saf* 2022; **240**: 113697.

65. Li M, Liu T, Yang T, Zhu J, Zhou Y, Wang M, et al. Gut microbiota dysbiosis involves in host non-alcoholic fatty liver disease upon pyrethroid pesticide exposure. *Environ Sci Ecotechnology* 2022; **11**: 100185.

66. Kubo S, Hirano T, Miyata Y, Ohno S, Onaru K, Ikenaka Y, et al. Sex-specific behavioral effects of acute exposure to the neonicotinoid clothianidin in mice. *Toxicol Appl Pharmacol* 2022; **456**: 116283.

67. Onaru K, Ohno S, Kubo S, Nakanishi S, Hirano T, Mantani Y, et al. Immunotoxicity evaluation by subacute oral administration of clothianidin in Sprague-Dawley rats. *J Vet Med Sci* 2020; **82**: 360–372.

68. Bao H, Gao H, Zhang J, Lu H, Yu N, Shao X, et al. Neonicotinoids stimulate H2-limited methane emission in *Periplaneta americana* through the regulation of gut bacterium community. *Environ Pollut* 2021; **285**: 117237.

69. Zhang Q, Gu S, Wang Y, Hu S, Yue S, Wang C. Stereoselective metabolic disruption of cypermethrin by remolding gut homeostasis in rat. *J Environ Sci* 2023; **126**: 761–771.

70. Liu Q, Shao W, Zhang C, Xu C, Wang Q, Liu H, et al. Organochloride pesticides modulated gut microbiota and influenced bile acid metabolism in mice. *Environ Pollut* 2017; **226**: 268–276.

71. Zhan J, Liang Y, Liu D, Ma X, Li P, Zhai W, et al. Pectin reduces environmental pollutant-induced obesity in mice through regulating gut microbiota: A case study of p,p′-DDE. *Environ Int* 2019; **130**: 104861.

72. Zhou S, Dong J, Liu Y, Yang Q, Xu N, Yang Y, et al. Effects of acute deltamethrin exposure on kidney transcriptome and intestinal microbiota in goldfish (*Carassius auratus*). *Ecotoxicol Environ Saf* 2021; **225**: 112716.

73. Dong ZX, Tang QH, Li WL, Wang ZW, Li X-J, Fu CM, et al. Honeybee (*Apis mellifera*) resistance to deltamethrin exposure by Modulating the gut microbiota and improving immunity. *Environ Pollut* 2022; **314**: 120340.

74. Wang H, Zhang C, Cheng P, Wang Y, Liu H, Wang H, et al. Differences in the intestinal microbiota between insecticide‐resistant and ‐sensitive Aedes albopictus based on full‐length 16S rRNA sequencing. *MicrobiologyOpen* 2021; **10**: e1177.

75. Tang J, Wang W, Jiang Y, Chu W. Diazinon exposure produces histological damage, oxidative stress, immune disorders and gut microbiota dysbiosis in crucian carp (*Carassius auratus gibelio*). *Environ Pollut* 2021; **269**: 116129.

76. Gao B, Bian X, Mahbub R, Lu K. Sex-specific effects of organophosphate diazinon on the gut microbiome and its metabolic functions. *Environ Health Perspect* 2017; **125**: 198–206.

77. Hua Q, Adamovsky O, Vespalcova H, Boyda J, Schmidt JT, Kozuch M, et al. Microbiome analysis and predicted relative metabolomic turnover suggest bacterial heme and selenium metabolism are altered in the gastrointestinal system of zebrafish (*Danio rerio*) exposed to the organochlorine dieldrin. *Environ Pollut* 2021; **268**: 115715.

78. Paris L, Peghaire E, Mone A, Diogon M, Debroas D, Delbac F, et al. Honeybee gut microbiota dysbiosis in pesticide/parasite co-exposures is mainly induced by *Nosema ceranae*. *J Invertebr Pathol* 2020; **172**: 107348.

79. Naggar YA, Singavarapu B, Paxton RJ, Wubet T. Bees under interactive stressors: the novel insecticides flupyradifurone and sulfoxaflor along with the fungicide azoxystrobin disrupt the gut microbiota of honey bees and increase opportunistic bacterial pathogens. *Sci Total Environ* 2022; **849**: 157941.

80. Rothman JA, Russell KA, Leger L, McFrederick QS, Graystock P. The direct and indirect effects of environmental toxicants on the health of bumblebees and their microbiomes. *Proc Biol Sci* 2020; **287**: 20200980.

81. Raymann K, Motta EVS, Girard C, Riddington IM, Dinser JA, Moran NA. Imidacloprid decreases honey bee survival rates but does not affect the gut microbiome. *Appl Environ Microbiol* 2018; **84**: e00545-18.

82. Hong Y, Huang Y, Wu S, Yang X, Dong Y, Xu D, et al. Effects of imidacloprid on the oxidative stress, detoxification and gut microbiota of Chinese mitten crab, *Eriocheir sinensis*. *Sci Total Environ* 2020; **729**: 138276.

83. Daisley BA, Trinder M, McDowell TW, Welle H, Dube JS, Ali SN, et al. Neonicotinoid-induced pathogen susceptibility is mitigated by *Lactobacillus plantarum* immune stimulation in a *Drosophila melanogaster* model. *Sci Rep* 2017; **7**: 2703.

84. Chmiel JA, Daisley BA, Burton JP, Reid G. Deleterious effects of neonicotinoid pesticides on *Drosophila melanogaster* immune pathways. *mBio* 2019; **10**: e01395-19.

85. Burke AP, Niibori Y, Terayama H, Ito M, Pidgeon C, Arsenault J, et al. Mammalian susceptibility to a neonicotinoid insecticide after fetal and early postnatal exposure. *Sci Rep* 2018; **8**: 16639.

86. Yan S, Tian S, Meng Z, Sun W, Xu N, Jia M, et al. Synergistic effect of ZnO NPs and imidacloprid on liver injury in male ICR mice: Increase the bioavailability of IMI by targeting the gut microbiota. *Environ Pollut* 2022; **294**: 118676.

87. Yang G, Yuan X, Jin C, Wang D, Wang Y, Miao W, et al. Imidacloprid disturbed the gut barrier function and interfered with bile acids metabolism in mice. *Environ Pollut* 2020; **266**: 115290.

88. Khalil SR, Awad A, Mohammed HH, Nassan MA. Imidacloprid insecticide exposure induces stress and disrupts glucose homeostasis in male rats. *Environ Toxicol Pharmacol* 2017; **55**: 165–174.

89. Fu Z, Han F, Huang K, Zhang J, Qin JG, Chen L, et al. Impact of imidacloprid exposure on the biochemical responses, transcriptome, gut microbiota and growth performance of the Pacific white shrimp *Litopenaeus vannamei*. *J Hazard Mater* 2022; **424**: 127513.

90. Receveur JP, Pechal JL, Benbow ME, Donato G, Rainey T, Wallace JR. Changes in larval mosquito microbiota reveal non-target effects of insecticide treatments in hurricane-created habitats. *Microb Ecol* 2018; **76**: 719–728.

91. Zhu L, Qi S, Xue X, Niu X, Wu L. Nitenpyram disturbs gut microbiota and influences metabolic homeostasis and immunity in honey bee (*Apis mellifera L*.). *Environ Pollut* 2020; **258**: 113671.

92. Yan S, Tian S, Meng Z, Yan J, Jia M, Li R, et al. Imbalance of gut microbiota and fecal metabolites in offspring female mice induced by nitenpyram exposure during pregnancy. *Chemosphere* 2020; **260**: 127506.

93. Yan S, Tian S, Meng Z, Teng M, Sun W, Jia M, et al. Exposure to nitenpyram during pregnancy causes colonic mucosal damage and non-alcoholic steatohepatitis in mouse offspring: The role of gut microbiota. *Environ Pollut* 2021; **271**: 116306.

94. Muturi EJ, Dunlap C, Smartt CT, Shin D. Resistance to permethrin alters the gut microbiota of *Aedes aegypti*. *Sci Rep* 2021; **11**: 14406.

95. Nasuti C, Fattoretti P, Carloni M, Fedeli D, Ubaldi M, Ciccocioppo R, et al. Neonatal exposure to permethrin pesticide causes lifelong fear and spatial learning deficits and alters hippocampal morphology of synapses. *J Neurodev Disord* 2014; **6**: 7.

96. Nasuti C, Coman MM, Olek RA, Fiorini D, Verdenelli MC, Cecchini C, et al. Changes on fecal microbiota in rats exposed to permethrin during postnatal development. *Environ Sci Pollut Res* 2016; **23**: 10930–10937.

97. Liu Y-J, Qiao N-H, Diao Q-Y, Jing Z, Vukanti R, Dai P-L, et al. Thiacloprid exposure perturbs the gut microbiota and reduces the survival status in honeybees. *J Hazard Mater* 2020; **389**: 121818.

98. Djellal D, Haddad S, Gasmi S, Chouit Z, Kebieche M, Hachemi M, et al. Chronic thiacloprid exposure impairs cognitive function and triggers mitochondrial apoptosis pathway in rat striatum and hippocampus: neuropreventive effect of bitter apricot kernels extract (*prunus armeniaca l*.). *J Microbiol Biotechnol Food Sci* 2022; e9089.

99. Fu Z, Han F, Huang K, Zhang J, Qin JG, Chen L, et al. Combined toxic effects of thiamethoxam on intestinal flora, transcriptome and physiology of Pacific white shrimp *Litopenaeus vannamei*. *Sci Total Environ* 2022; **830**: 154799.

100. Yang F-W, Fang B, Pang G-F, Zhang M, Ren F-Z. Triazophos and its metabolite diethyl phosphate have different effects on endocrine hormones and gut health in rats. *J Environ Sci Health Part B* 2021; **56**: 566–576.

101. Zhang Q, Zhu D, Ding J, Zheng F, Zhou S, Lu T, et al. The fungicide azoxystrobin perturbs the gut microbiota community and enriches antibiotic resistance genes in Enchytraeus crypticus. *Environ Int* 2019; **131**: 104965.

102. Zhang Q, Yu Y, Jin M, Deng Y, Zheng B, Lu T, et al. Oral azoxystrobin driving the dynamic change in resistome by disturbing the stability of the gut microbiota of *Enchytraeus crypticus*. *J Hazard Mater* 2022; **423**: 127252.

103. Wang K, Chen H, Fan R-L, Lin Z-G, Niu Q-S, Wang Z, et al. Effect of carbendazim on honey bee health: Assessment of survival, pollen consumption, and gut microbiome composition. *Ecotoxicol Environ Saf* 2022; **239**: 113648.

104. Jin Y, Zeng Z, Wu Y, Zhang S, Fu Z. Oral exposure of mice to carbendazim induces hepatic lipid metabolism disorder and gut microbiota dysbiosis. *Toxicol Sci* 2015; **147**: 116–126.

105. Jin C, Zeng Z, Wang C, Luo T, Wang S, Zhou J, et al. Insights into a possible mechanism underlying the connection of carbendazim-induced lipid metabolism disorder and gut microbiota dysbiosis in mice. *Toxicol Sci* 2018; **166**: 382–393.

106. Song J, Li T, Zheng Z, Fu W, Long Z, Shi N, et al. Carbendazim shapes microbiome and enhances resistome in the earthworm gut. *Microbiome* 2022; **10**: 63.

107. Wu T, Han B, Wang X, Tong Y, Liu F, Diao Q, et al. Chlorothalonil alters the gut microbiota and reduces the survival of immature honey bees reared in vitro. *Pest Manag Sci* 2022; **78**: 1976–1981.

108. Zhang H, Yang G, Bao Z, Jin Y, Wang J, Chen J, et al. Stereoselective effects of fungicide difenoconazole and its four stereoisomers on gut barrier, microbiota, and glucolipid metabolism in male mice. *Sci Total Environ* 2022; **805**: 150454.

109. Jiang J, Chen L, Wu S, Lv L, Liu X, Wang Q, et al. Effects of difenoconazole on hepatotoxicity, lipid metabolism and gut microbiota in zebrafish (Danio rerio). *Environ Pollut* 2020; **265**: 114844.

110. Xu C, Liu Q, Huan F, Qu J, Liu W, Gu A, et al. Changes in gut microbiota may be early signs of liver toxicity induced by epoxiconazole in rats. *Chemotherapy* 2014; **60**: 135–142.

111. Kaziem AE, He Z, Li L, Wen Y, Wang Z, Gao Y, et al. Changes in soil and rat gut microbial diversity after long-term exposure to the chiral fungicide epoxiconazole. *Chemosphere* 2021; **272**: 129618.

112. Jin C, Zeng Z, Fu Z, Jin Y. Oral imazalil exposure induces gut microbiota dysbiosis and colonic inflammation in mice. *Chemosphere* 2016; **160**: 349–358.

113. Jin C, Xia J, Wu S, Tu W, Pan Z, Fu Z, et al. Insights into a possible influence on gut microbiota and intestinal barrier function during chronic exposure of mice to imazalil. *Toxicol Sci* 2018; **162**: 113–123.

114. Wang X, Hu L, Wang C, He B, Fu Z, Jin C, et al. Cross-generational effects of maternal exposure to imazalil on anaerobic components and carnitine absorption associated with OCTN2 expression in mice. *Chemosphere* 2022; **308**: 136542.

115. Jin C, Luo T, Zhu Z, Pan Z, Yang J, Wang W, et al. Imazalil exposure induces gut microbiota dysbiosis and hepatic metabolism disorder in zebrafish. *Comp Biochem Physiol Part C Toxicol Pharmacol* 2017; **202**: 85–93.

116. Meng Z, Liu L, Jia M, Li R, Yan S, Tian S, et al. Impacts of penconazole and Its enantiomers exposure on gut microbiota and metabolic profiles in mice. *J Agric Food Chem* 2019; **67**: 8303–8311.

117. Morgan AM, Hassanen EI, Ogaly HA, Al Dulmani SA, Al-Zahrani FAM, Galal MK, et al. The ameliorative effect of N-acetylcysteine against penconazole induced neurodegenerative and neuroinflammatory disorders in rats. *J Biochem Mol Toxicol* 2021; **35**: e22884.

118. Jin C, Weng Y, Zhang Y, Bao Z, Yang G, Fu Z, et al. Propamocarb exposure has the potential to accelerate the formation of atherosclerosis in both WT and ApoE−/− mice accompanied by gut microbiota dysbiosis. *Sci Total Environ* 2021; **800**: 149602.

119. Wu S, Jin C, Wang Y, Fu Z, Jin Y. Exposure to the fungicide propamocarb causes gut microbiota dysbiosis and metabolic disorder in mice. *Environ Pollut* 2018; **237**: 775–783.

120. Wu S, Luo T, Wang S, Zhou J, Ni Y, Fu Z, et al. Chronic exposure to fungicide propamocarb induces bile acid metabolic disorder and increases trimethylamine in C57BL/6J mice. *Sci Total Environ* 2018; **642**: 341–348.

121. Zhang R, Pan Z, Wang X, Shen M, Zhou J, Fu Z, et al. Short-term propamocarb exposure induces hepatic metabolism disorder associated with gut microbiota dysbiosis in adult male zebrafish. *Acta Biochim Biophys Sin* 2019; **51**: 88–96.

122. Meng Z, Huang S, Sun W, Yan S, Chen X, Diao J, et al. A typical fungicide and its main metabolite promote liver damage in mice through impacting gut microbiota and intestinal barrier function. *J Agric Food Chem* 2021; **69**: 13436–13447.

123. Hu L, Wang X, Bao Z, Xu Q, Qian M, Jin Y. The fungicide prothioconazole and its metabolite prothioconazole-desthio disturbed the liver-gut axis in mice. *Chemosphere* 2022; **307**: 136141.

124. Meng Z, Sun W, Liu W, Wang Y, Jia M, Tian S, et al. A common fungicide tebuconazole promotes colitis in mice via regulating gut microbiota. *Environ Pollut* 2022; **292**: 118477.

125. Song Y, Shi J, Xiong Z, Shentu X, Yu X. Three antimicrobials alter gut microbial communities and causing different mortality of brown planthopper, Nilaparvata lugens Stål. *Pestic Biochem Physiol* 2021; **174**: 104806.

126. Kong A, zhang C, Cao Y, Cao Q, Liu F, Yang Y, et al. The fungicide thiram perturbs gut microbiota community and causes lipid metabolism disorder in chickens. *Ecotoxicol Environ Saf* 2020; **206**: 111400.

127. Zhang Y, Ding Y, Mo Q, Kulyar MF-E-A, He Y, Yao W, et al. Sodium butyrate ameliorates thiram-induced tibial dyschondroplasia and gut microbial dysbiosis in broiler chickens. *Ecotoxicol Environ Saf* 2022; **245**: 114134.

128. Vieira R, Venâncio C, Félix L. Teratogenic, oxidative stress and behavioural outcomes of three fungicides of natural origin (Equisetum arvense, Mimosa tenuiflora, thymol) on zebrafish (*Danio rerio*). *Toxics* 2021; **9**: 8.

129. He S, Yu D, Li P, Zhang M, Xing S, Liu B, et al. A new perspective on endocrine disrupting effects of triphenyltin on marine medaka: From brain transcriptome, gut content metabolome and behavior. *Chemosphere* 2022; **307**: 136190.

130. Zhang S-Q, Li P, He S-W, Xing S-Y, Cao Z-H, Zhao X-L, et al. Assessing the ecotoxicity of combined exposure to triphenyltin and norfloxacin at environmental levels: A case study of immunotoxicity and metabolic regulation in carp (*Cyprinus carpio*). *Chemosphere* 2023; **313**: 137381.
